# Improvement of the Mechanical Properties of Silica Aerogels for Thermal Insulation Applications through a Combination of Aramid Nanofibres and Microfibres

**DOI:** 10.3390/gels9070535

**Published:** 2023-06-30

**Authors:** Mariana Emilia Ghica, Jandira G. S. Mandinga, Teresa Linhares, Cláudio M. R. Almeida, Luisa Durães

**Affiliations:** University of Coimbra, CIEPQPF, Department of Chemical Engineering, 3030-790 Coimbra, Portugal; jandiramandinga2014@outlook.com (J.G.S.M.); tlinhares@eq.uc.pt (T.L.); claudio@eq.uc.pt (C.M.R.A.)

**Keywords:** silica aerogels, nanofibers, thermal insulation, scale-up, space compliance

## Abstract

Reinforcement of silica aerogels, remarkable lightweight mesoporous materials with outstanding insulation performance, is still a challenging research topic. Among the strategies used to overcome their brittleness, one of the most effective is the manufacturing of aerogel composites with embedded fibres. In this work, the incorporation of nanofibres together with microfibres in a tetraethoxysilane–vinyltrimethoxysilane matrix is investigated for the first time for the development of novel aerogel nanocomposites. The nanofibres, synthesized from different aramid fibres, including Kevlar^®^ pulp, Technora^®^, Teijinconex^®^ and Twaron^®^ fibres, were used in different combinations with microaramids and the resulting nanocomposites were thoroughly investigated for their physicochemical and thermomechanical features. The properties depended on the type and amount of the nano/microfibre used. While the microfibres exhibited low interaction with the silica matrix, the higher surface of the nanofibres ensured increased contact with the gel matrix. A low bulk density of 161 kg m^−3^ and thermal conductivity of 38.3 mW m^−1^ K^−1^ (Hot Disk^®^) was achieved when combining the nanofibres obtained from Kevlar^®^ pulp with the Technora^®^ or Teijinconex^®^ long fibres. The nanofibres showed higher dispersion and random orientation and in combination with microfibres led to the improvement by a factor of three regarding the mechanical properties of the aerogel nanocomposites reinforced only with microfibres. The scale-up process of the samples and simulated tests of thermal cycling and vacuum outgassing successfully conducted indicate good compliance with space applications.

## 1. Introduction

Thanks to their high porosity (>90%), nanoporous structure, and crosslinked network, silica aerogels exhibit thermal conductivity values closer to or even lower than air (0.021 W m^−1^ K^−1^ at 0 °C under 1 bar) [1]. This property ensures their use as distinguished thermal insulation materials in a wide range of applications, among which are buildings, protective suits, aerospace, and chemical engineering [1,2]. Despite this, for applications that require monoliths, silica aerogels are not appropriate, since their mechanical properties are poor, including high brittleness and low strength [2]. Mechanical reinforcing strategies, the topic of diverse reviews [3], include careful selection of the silane precursor, a combination of the inorganic silica with flexible organic polymers, the introduction of fibre networks, etc. Among the methods used to strengthen silica networks, the incorporation of fibres and nanofibres into silica aerogels is one of the most efficient [4,5]. Through interaction with the gel matrix, the fibres avoid the collapse of the structure that may occur during drying [4] and, thus, preserves their monolithic form. The use of inorganic fibres, such as glass fibres, has proven their efficacity for the structural reinforcement of aerogels; however, they do not proffer high strength, and they might increase the aerogel’s density [6]. On the other hand, the use of organic fibres was shown to have good compatibility with the silica sol and confer increased flexibility and reduced shrinkage during drying [4,7]. Several organic fibres were used to fortify silica aerogels, including carbon fibres [8], cellulose-based, such as cotton [9], flax [10], nanocellulose [11], or polymer-based, such as poly(p-phenylene terephthalamide) [6,12], polypropylene [13], polyvinyl alcohol [14], etc. Among them, the poly(p-phenylene terephthalamide), known as polyamide or aramid fibres, with low density, high mechanical strength, elevated thermal stability and nonflammable character [15] represent one of the best choices for application in forefront fields, such as electronics, tanks insulation, aerospace, bulletproof products, etc. [16,17]. Despite the effectiveness of the mechanical reinforcement these fibres may grant, their diameter is much higher than the pore sizes of the silica network and, hence, may result in an unbalanced response when submitted to stress loads [4]. To increase the interconnectivity of components in the three-dimensional aerogel network, which is essential for stress transfer, nanoscale components, such as nanofibers, may be introduced [18]. The nanofibers, with a smaller diameter and higher surface area than microfibres, enhance the contact with the gel matrix at the microscopic level and, thus, improve the mechanical resistance of the resulting aerogels. The classical methods for nanofibres preparation, such as phase separation and self-assembly, are not suitable for aramid nanofibre (ANF) synthesis, due to the low efficiency and low quality of nanofibers [19]. Novel strategies for the synthesis of ANF include (i) bottom-up methods, such as induced polymerisation and electrospinning and (ii) top-down methods, such as deprotonation and mechanical disintegration [19]. There are few reports on silica aerogels reinforced with nanofibres, including attapulgite [5], cellulose [11], silica [20] and polymer [21,22] nanofibres, but none of them used aramid nanofibres. Since Kotov’s group pioneered the preparation of an aramid aerogel from branched aramid nanofibres and dried it by supercritical drying [18], the self-assembled nanofiber-based aerogels aroused the great interest of researchers [23,24]. These aerogels are usually dried using supercritical or freeze-drying techniques. They were also prepared containing MOFs [25], graphene/polymers [26], and carbon nanotubes [27]. However, the reported ANF aerogels are still facing poor mechanical strength and weak structure stability due to randomly packed ANF forming an almost 3D network with a weak interfibre connection [24].

In this sense, to improve the mechanical strength of aerogels, we report here, for the first time, the use of ANF to reinforce silica aerogels. As far as we know, there are no studies reporting on ANF-reinforced silica aerogels, except one work in which aerogels prepared from ANF were coated with a silica shell by chemical vapour deposition and the composites were dried by CO_2_ supercritical drying [28]. Herein, we used a mixture of aramid fibres (AF) and ANF to boost the strength of the silica aerogels which were dried by ambient pressure, a safer and less expensive process than supercritical drying. The aramid nanofibres were produced by deprotonation and their size was estimated by different methods, including dynamic light scattering (DLS) and scanning electron microscopy (SEM). Several combinations of AF and ANF were used and the influence of the amount of nanofibres and microfibres on the bulk density and thermal conductivity of the nanocomposites was investigated. The combinations with the best performance were tested regarding their thermal stability and mechanical resistance. The correlation of the nanocomposites’ thermal and mechanical features with their structural properties is discussed. Their practical application was verified through simulated tests in conformity with space conditions and the scale-up process of the optimized nanocomposites was also performed.

## 2. Results and Discussion

### 2.1. Characterisation of the Aramid Nanofibres

The nanofibres, synthesized from different aramid fibres (Kevlar^®^ pulp—KP, Technora^®^—Tch, Teijinconex^®^—Teij, and Twaron^®^—Tw), were evaluated regarding their chemical, structural, and morphological features; the characterisation was performed by FTIR and SEM for the dried residues and dynamic light scattering (DLS) was also applied to dimethylsulfoxide (DMSO) suspension of the fibres.

#### 2.1.1. SEM Analysis of AF and ANF

A comparative study of the size and morphology of aramid fibres (KP, Tch, Teij, Tw) and corresponding nanofibres (NKP, NTch, NTeij, and NTw) was carried out by scanning electron microscopy (Figure S1). The SEM images revealed that the fibres are gathered in bunches, the short ones, KP, were ribbon shaped and much thinner than the others, with diameters of 1.1 ± 0.2 μm (Figure S1(a1)), while the long AF (Tch, Teij, Tw—Figure S1(b1)–(d1)) were cylindrical shape with diameters: 8.7 ± 0.8 μm (Tw), 11.8 ± 1.0 μm (Teij), 13.3 ± 1.2 μm (Tch). Regarding the nanofibres, in the case of NTeij and NTch, it was difficult to visualize them (Figure S1(c2,d2)), probably due to their small size or low loading in the analyzed sample. However, for NKP and NTw (Figure S1(a2,b2)), it was possible to observe that the reduced-size fibrils were obtained when compared with the fibre counterpart. The estimated diameters were 251 ± 15 nm (NKP), 205 ± 10 nm (NTch), and for NTw a mixture of wider (542 ± 25 nm diameter) and thinner (247± 10 nm diameter) fibres was observed. It was not possible to estimate the size of the NTeij from the SEM images. The size of the NKP obtained here is similar to those reported in [24,28] also from Kevlar.

#### 2.1.2. DLS Analysis of Nanofibres

**Figure 1:**
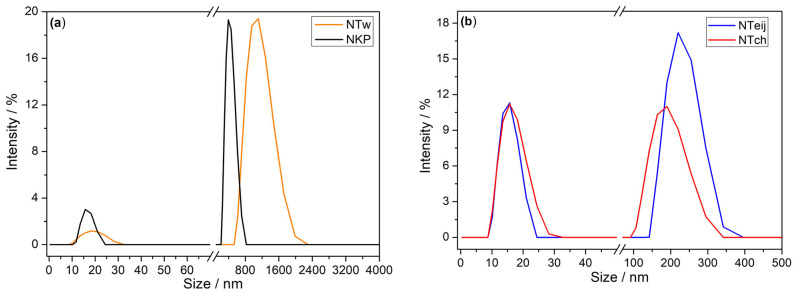
Intensity-weighted nanofibre size distributions for (**a**) NKP and NTw and (**b**) NTch and NTeij dispersions in DMSO.

Dynamic light scattering is generally used to determine the size of nanoparticles. However, since the SEM images did not permit a very accurate determination of the nanofiber’s size, DLS was also used to estimate the dimensions of the nanofibres. In Figure 1 are illustrated the plots obtained during DLS measurement. As observed, all the samples contain a nonhomogeneous mixture of submicron and nanofibres with a bimodal distribution of weighted intensity.

For NKP and NTw, the highest intensity peak of light appears at 431 nm and 1095 nm, indicating these are the predominant size of nanofibres, smaller nanofibres with diameters around 20 nm are also present. The NTch and NTeij showed a similar distribution of the intensity weighted size; the lowest intensity peak was around 15 nm and the highest intensity peak at 180 and 220 nm, respectively. Therefore, the NTch and NTeij are much smaller than NKP and NTw, confirming the observations during the filtration process, which did not allow for the obtaining of any filtrate for NTch and NTeij under the conditions used. The values obtained are in line with those obtained by SEM. Due to this practical aspect, only the NKP and NTw were further used in combination with AF to reinforce the silica gels.

#### 2.1.3. FTIR Analysis of Nanofibres

**Figure 2:**
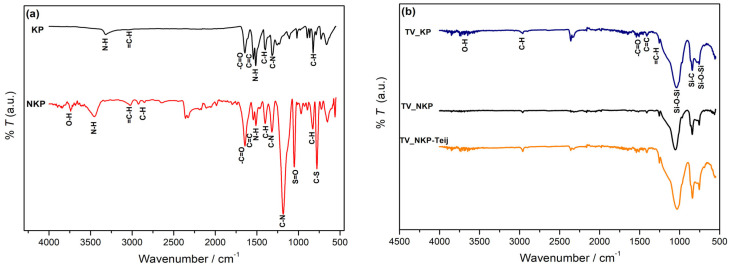
ATR-FTIR spectra of (**a**) fibres and (**b**) aerogel composites based on tetraethoxysilane/vinyltrimethoxysilane (TV) with different reinforcement combinations (nomenclature in Table S1).

Figure 2a shows a comparison between the ATR-FTIR spectra of the Kevlar pulp (KP) and the corresponding nanofibres, NKP. For both samples, the following typical vibration bands of aramids were observed [12]: at 3313 cm^−1^ (KP) and 3442 cm^−1^ (NKP), associated with stretching of -NH, at 3020 cm^−1^, corresponding to stretching of =C-H of the aromatic ring, at 1643 cm^−1^ and 1606 cm^−1^, representing the stretching of carbonyl (-C=O) in the amide I and C=C of the aromatic ring, at 1513 cm^−1^, attributed to the -NH bending of the amide II, at 1398 cm^−1^, associated to the in-plane bending of -C-H, at 1200–1316 cm^−1^, corresponding to asymmetrical stretching of the -C-N bonds of the amide III, at 822 cm^−1^ due to out of plane bending of -C-H of the aromatic ring. The successful synthesis of nanofibres is proved by (i) the shift of the -NH vibration band in NKP towards a higher value, clearly indicating the break of the H-bonded N-H and(ii) the increase of intensity for the N-H and C=O stretching bands in the case of NKP, evidencing the presence of free amine and carbonyl groups [29]. Other differences between the NKP and KP spectra were observed. One is the appearance of the OH stretching vibration at 3739 cm^−1^, indicating most probably the adsorption of water by the nanofibres. Additionally, there are several vibration bands in the NKP’s spectrum revealing the presence of DMSO, namely at 2850 cm^−1^ associated with the stretching of -C-H of the methyl group in DMSO [30], at 1048 cm^−1^, due to the stretching vibration of S=O bonds [31], and at 780 cm^−1^, attributed to the vibration of -C-S bond [30].

The band around 2300 cm^−1^ is most likely due to the CO_2_ interference in the analysis, also confirmed by the very small peaks near 4000 cm^−1^.

### 2.2. Characterisation of the Reinforced Silica Aerogels

#### 2.2.1. Visual Aspect of the Nanocomposites

**Figure 3:**
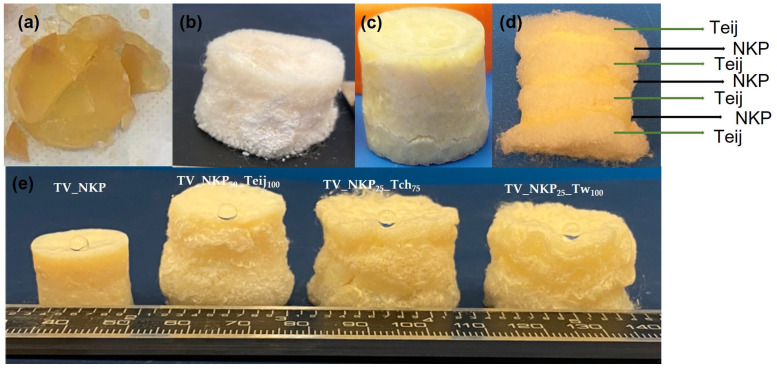
Visual aspect of the silica aerogels containing (**a**) NKP_DMSO_, (**b**) NKP_DMSO_/Teij, (**c**) NKP, (**d**) NKP/Teij, and (**e**) aerogel composites with different fibre combinations (nomenclature in Table S1).

Different aerogel composites were prepared in this work, their nomenclature is presented in Table S1. Taking into consideration the ability of ANF to self-assemble and form a gel, we first attempt to prepare aerogels by directly adding the ANF dispersion in DMSO to the silica precursors; the sol turned into a gel in a few hours. However, these gels were very fragile and broke during the ageing step (Figure 3a). In a different approach, aramid fibres were added to the same composition to fortify the network. The final dried aerogels were monoliths, however, easily loosening material due to fragmentation (Figure 3b). Therefore, it was decided to separate the nanofibres from the DMSO phase, filtration being used, and then add the fibres to the silica sol. Thus, the aerogels that will be further described include silica with either nanofibres (NKP and NTw), fibres (KP, Tch, Teij, Tw), or their combinations, the microfibres-based ones being prepared for reference use.

The aerogels prepared only with nanofibres were very rigid and could easily break (Figure 3c), while those containing a combination of ANF and AF were much more flexible and permitted the observation of the layers of fibres inside (Figure 3d). In Figure 3e are shown the pictures of aerogel composites with different fibre combinations. It is clear that the aerogels containing only nanofibres suffered a higher shrinkage; this being also visible in the nanofibre layer of the composite with AF and ANF combinations (Figure 3d).

#### 2.2.2. Gelation Time of the Nanocomposites

In Table S2 are presented the gelation times for different aerogel samples. Generally, it was observed that the gelation time depends both on microfibres and nanofibres loading, increasing with the increase of AF, while decreasing when the amount of ANF was increased. However, this behaviour is not always verified, mainly due to the uncertainty of time measurement, as well as because of the system’s heterogeneity. On the one hand, the microfibres hamper the formation of the silica network, due to steric hindrance. On the other hand, the nanofibres suffer swelling, thus leading to a decrease in the volume of solvent available for precursor dilution. Consequently, the local concentration of precursors increases, leading to a decrease of the gelation time, since the species in the solution are closer. At the same time, the nanofibres have higher interaction with the sol, constituting nucleation sites of silica, thence contributing to the decrease of the gelation time.

#### 2.2.3. Chemical Characterisation of the Nanocomposites

The chemical bonding in aerogel samples containing different reinforcements was achieved by ATR-FTIR, Figure 2b; no significant differences were observed between the spectra. Generally, the vibration bands, common for all spectra were observed at ~1600 cm^−1^ and 1409 cm^−1^, attributed to stretching of C=C and bending of =C-H present in vinylic groups, 1213 cm^−1^, related with symmetric bending of -C-H, at 1025–1049 cm^−1^, due to asymmetric stretching vibration of Si-O-Si, at 841, related with stretching of Si-C, at 754 cm^−1^, associated to symmetric stretching of Si-O-Si [12,32]. The occurrence of small bands at ~3313 cm^−1^ and at ~1643 cm^−1^, is related to stretching vibrations of -NH and -C=O of the amide group, indicating the presence of fibres in the matrix [12]. Finally, the surface modification with the silylation agent HMDZ is confirmed by the presence of the band at 2958 cm^−1^, attributed to the symmetrical stretching vibration of -C-H in the Si-CH_3_ group [12].

All the samples have a hydrophobic character (Figure 3e), with contact angles between 127° and 142°. These results are in agreement with the FTIR analysis, indicating a successful modification of the nanofibre/microfibre–silica network by the HMDZ, and a high number of methyl groups attached to the composite aerogels.

#### 2.2.4. SEM Analysis of the Nanocomposites

**Figure 4:**
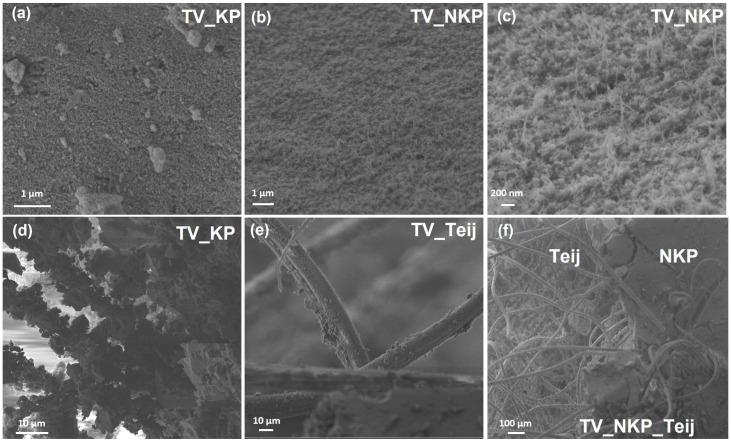
Scanning electron microscope images of different aerogel composites (nomenclature in Table S1) showing: (**a**–**c**) aerogel matrix, (**d**–**f**) interaction of the fibres with the aerogel matrix. Reinforcement was performed with: (**a**,**d**) KP, (**b**,**c**) NKP, (**e**) Teij, and (**f**) NKP and Teij.

In Figure 4a–c are illustrated the scanning electron microscope images obtained at the surface of the aerogel composites reinforced with KP and NKP. The structure of the aerogel is similar, a porous interconnected 3D matrix; in Figure 4c, with higher magnification, it is possible to observe the NKP fibrils with diameters of 18.6 ± 2.5 nm, in agreement with the smaller value obtained by DLS.

The interaction between aramid fibres and silica was previously reported to occur through interfacial adhesion [6]. The fibres represent a support for silica-gel particles to attach and grow. The interaction of fibres with the aerogel matrix can be observed in Figure 4d–f. The short KP fibres had higher interaction with the matrix and the aerogel grows around the fibres (Figure 4d). In the case of long fibres, the Teij have low interaction with the aerogel matrix (Figure 4e), as only a small amount of gel is observed around them. In the sample reinforced with microfibres and nanofibres (Figure 4f), it was observed that there is a clear interface created by the layer with AF and ANF. The preferential growth of silica around ANF might be explained by the probable hydrogen bonding between the hydroxyl groups of silica and the free amine and carbonyl groups present in nanofibres.

#### 2.2.5. Density, Linear Shrinkage, and Porosity of the Nanocomposites

The linear shrinkage and bulk density values of the aerogels synthesized with NKP and NTw combinations are presented in Table 1. The linear shrinkage is dictated by the type of microfibre used, being higher in the combinations with Teij reinforcement, as previously observed when using only aramid fibres [12]; the behaviour was not influenced by the addition of nanofibres, indifferently of their type.

The influence of the nanofiber content on the bulk-density value is illustrated in Figure S2. Generally, the aerogels prepared with NKP (Figure S2a) had lower bulk-density values than those prepared with NTw (Figure S2b). The combinations of nanofibres with microfibres led to more homogeneous nanocomposites when using the mixtures with Teij; however, the bulk-density values of these aerogels were higher, compared with those obtained with mixtures with Tch and Tw (Table 1). This was expected since bulk density is related to the linear shrinkage; therefore, similar behaviours were observed. The values of the aerogel composites were comprised between 161–317 kg m^−3^, comparable with those of the ANF aerogels coated with Si dried by supercritical CO_2_ (96–298 kg m^−3^) [28]. The lowest value of bulk density (161 kg m^−3^) was obtained with the NKP and Technora^®^ combination, which presents the lowest shrinkage (16.4%).

The calculated values of porosity were between 77.3 and 88.5%, the lowest values being obtained with the samples containing NKP and Teij, as expected since this behaviour was previously observed in [12] with aerogels reinforced only with long aramid fibres. The values are lower than those obtained only with AF and this is due to the presence of nanofibres filling the pores.

#### 2.2.6. Thermal Conductivity of the Nanocomposites

The total thermal conductivity for nanoporous materials is the sum of four terms: the conduction through the solid phase, the conduction through the gaseous phase, the thermal radiation, and the convection within pores, being this last negligible for aerogels [33]. The solid conductivity is that of the skeleton, the gaseous conductivity is related to the confinement of the gas in the aerogel’s pores and the radiation’s contribution depends on the density and pore size. Therefore, in order to understand the results obtained, it is necessary to analyze all these contributions.

The thermal conductivity values of the aerogels reinforced with combinations of nanofibers and microfibres are higher (Table 1) compared to their counterparts reinforced with only microfibres (32.5 mW m^−1^ K^−1^—Teij, 39.0 mW m^−1^ K^−1^—Tch, 50.8 mW m^−1^ K^−1^—Tw). This is a result of the decrease in porosity and increase in density with the addition of nanofibres (Table 1). It was shown that porosity is related to density and there is a relationship between density and thermal conductivity in silica aerogels [34], leading to higher thermal conductivity values with the increase of density. The values of thermal conductivity tend to decrease with the increase of microfibre content for the same amount of nanofibre. This is related to the decrease in density observed for these systems and the consequent increase in porosity.

**Figure 5:**
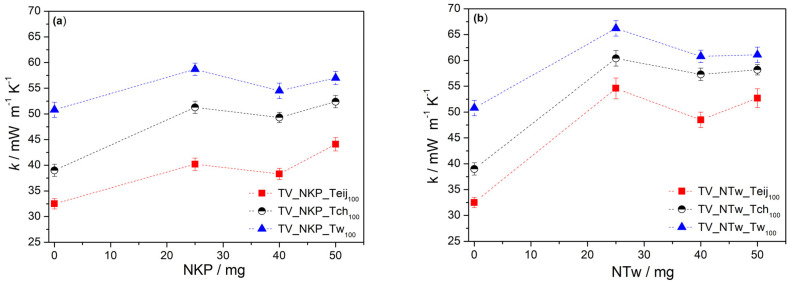
Influence of the quantity of (**a**) NKP and (**b**) NTw on the thermal conductivity of the nanocomposites (nomenclature in Table S1), considering equal quantities of different microfibres.

A representation of the thermal conductivity values for different concentrations of nanofibers (NKP and NTw) when keeping the same amount of microfibre, 100 mg, is shown in Figure 5. There is a small variation between the values among these nanocomposites with different amounts of nanofibres, with a slight decrease when using 40 mg of nanofibres (Figure 5a,b). This behaviour can be explained if considering two facts. On the one hand, the thermal conductivity is related to linear shrinkage, meaning that higher shrinkage led to smaller pores and lower gaseous thermal conductivity is achieved, due to the Knudsen effect [33]. On the other hand, the increase of solid phase (fibres) contributes to the increase of bulk density and sample densification leads to higher thermal conductivity values. For these reasons, there is a minimum value of thermal conductivity; however, when increasing the amount of nanofibers, the connectivity with the silica network increases; thus, the thermal conductivity values increase again.

Generally, the systems with NTw (Figure 5b) show higher thermal conductivity values, compared with those with NKP (Figure 5a). Among these last nanocomposites, the highest value of thermal conductivity was obtained in combination with Tw (58.7 mW m^−1^ K^−1^) and the lowest when using Teij (38.3 mW m^−1^ K^−1^). This behaviour is in agreement with the results obtained when only microfibres were used for the reinforcement of silica aerogels [12], indicating that the tendency is dictated by the microfibres. A comparison of the thermal conductivity values of the samples prepared here with those of similar systems in literature, namely aerogels prepared from aramid nanofibres or silica aerogels reinforced with aramid fibres or different nanofibers, is shown in Table S3. The aerogels in this work achieved attracting thermal conductivities when they were prepared by less complex methods. The lowest value obtained here is not as low (23.7–26.3 mW m^−1^ K^−1^) as that in [24] with an ANF aerogel prepared by directional freezing but is comparable with that of the ANF aerogel prepared by wet-spinning combined with freeze-drying (34 mW m^−1^ K^−1^) [23], as well as with that of the ANF aerogels coated with Si dried by supercritical CO_2_ (30–40 mW m^−1^ K^−1^) [28]. Similar values, 33.9 mW m^−1^ K^−1^ [35], 41.1 mW m^−1^ K^−1^ [36] and even higher, 71.8 mW m^−1^ K^−1^ [35], 89.3 mW m^−1^ K^−1^ [36] than the highest value registered here (66.2 mW m^−1^ K^−1^) were obtained with an ANF aerogel prepared by a modified freeze-drying method [35] and with high density fibrous polyimide sponges prepared by “self-gluing” concept [36]. Lower thermal conductivity values were obtained with the aerogel nanocomposites prepared herein under vacuum conditions (~900 mbar); the values dropped from 38.3 mW m^−1^ K^−1^ to 30.6 mW m^−1^ K^−1^ with a nanocomposite containing NKP and Teij and from 49.3 mW m^−1^ K^−1^ to 37.6 mW m^−1^ K^−1^ with an NKP and Tch aerogel.

Considering these results (higher bulk density and higher thermal conductivity), the aerogels containing NTw, indifferently of the microfibre used in combination, as well as those containing NKP and Tw fibre, will be no longer investigated concerning thermal stability and mechanical properties.

#### 2.2.7. Thermal Stability of the Nanocomposites

**Figure 6:**
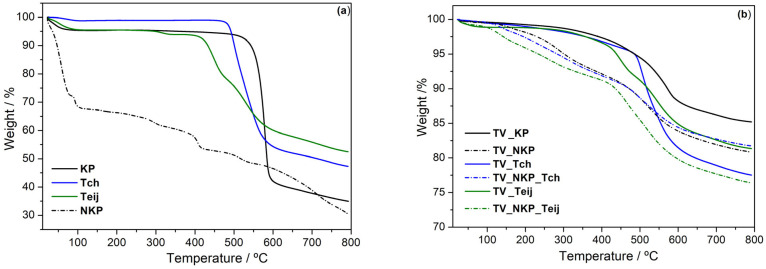
Thermograms for (**a**) AF and NKP and (**b**) silica aerogels reinforced with AF, NKP, and their combination (nomenclature as in Table S1).

The nanocomposites were investigated regarding their thermal stability using thermogravimetry (Figure 6b). For better comprehension, a study of the thermal stability of the fibres was also performed (Figure 6a). The results of the thermograms shown in Figure 6 are presented in Table S4.

When comparing the thermal stability of the microfibres, it is obvious that the one with higher stability is KP since it is a *para*-aramid, while Teij had the lowest stability, as expected, for being a *meta*-aramid; Tch is between them as it is a mixture of *meta*- and *para-*aramid (Table S4). The weight loss process of the microfibres is mainly due to the evaporation of water adsorbed by the fibres (up to 100 °C) and fibre degradation (above 400 °C), which occurs in two steps in the case of Tch and Teij [12]. On the other hand, the thermal phenomena observed for NKP are more complex than for KP, having associated the evaporation of water adsorbed from the deprotonation reaction (below 100 °C), but also DMSO evaporation (~250 °C). Additionally, the NKP degradation occurs in two steps (400 °C and 560 °C), distinct from KP. All these phenomena led to lower thermal stability of the NKP up to 550 °C, compared with any microfibres; however, the residue does not differ much from that of KP (Table S4), since most of the mass loss is not due to fibre degradation but it is associated with solvents evaporation.

From the thermogravimetric analysis of the nanocomposites, it is possible to conclude that all the aerogels can be used up to 550 °C, without significant weight loss; the residues were between 76.4–90%. There is only a small difference between the thermal stability at 550 °C of the aerogels reinforced with either KP or NKP and higher thermal stability is obtained when using Teij or Tch, especially for the latter due to the *para* configuration in its structure. This means that inside the silica network, the AF are less exposed to degradation. In combination with NKP, this behaviour is maintained; however, the thermal performance of the aerogels decreased due to the presence of solvents in the nanofibers, as previously observed. The residual mass of the aerogels reinforced with nanofibre was 76.4% when combined with Teij and 81.6% when combined with Tch.

#### 2.2.8. Mechanical Resistance of Aerogels

The curves obtained from the destructive test of silica composites reinforced with combinations of NKP and AF (Figure S3) illustrate the typical three regions-like behaviour, similar to the AF-silica aerogels [12]. When comparing the stress–strain curves for different samples, it was observed that the aerogels with NKP and Teij or Tch begin to densify at a lower strain (>50%) than that of AF-based aerogels (>70%), which is probably because the aerogel matrix is mainly concentrated in the region with nanofibers (as observed from SEM) and already presents higher bulk density due to higher shrinkage. The samples reinforced with Teij are more rigid than those with Tch and the tendency is maintained with the addition of nanofibres. The maximum stress, 10.2 MPa, was attained at 71% for the nanocomposite with NKP and Teij, and 8.9 MPa at 77% for the aerogel with NKP and Tch. These values are higher than the 5.1 MPa reached at a compressive strain of 70% by the ANF aerogel prepared by a modified freeze-drying method [35] and ANF aerogels reinforced with carbon nanotubes that reached not more than 1.23 MPa at 80% compressive strain [27]. A more detailed comparison with similar systems from the literature can be seen in Table S3. It was observed that the aerogels with very low densities [11,24,37], although presenting lower thermal conductivities [11,24], were less stress resistant.

**Figure 7:**
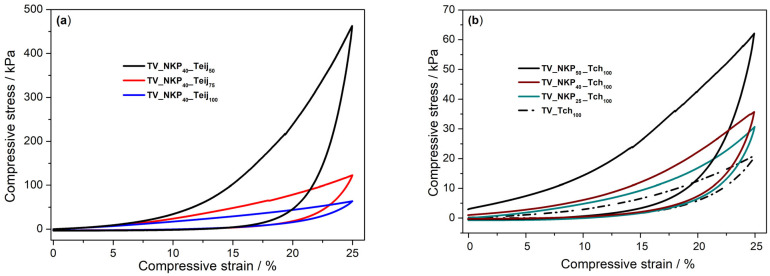
Compression–decompression curves for different systems containing (**a**) 40 mg NKP in combination with different amounts of Teij and (**b**) 100 mg Tch in combination with different amounts of NKP (nomenclature in Table S1).

Compression–decompression tests were carried out up to 25% strain and the influence of microfibre and nanofibre on the behaviour of nanocomposites was investigated (Figure 7). The Young’s modulus and recovery for the different systems are presented in Table 2.

The compression–decompression curves for the systems containing NKP and different amounts of Teij are illustrated in Figure 7a. It was observed that the increase in fibre quantity led to more flexible samples (the value of Young’s modulus decreases) for the same amount of NKP (Table 2). The same tendency was observed for the aerogels containing NKP and different amounts of Tch (not shown). This behaviour was expected since the long fibres support the macrostructure of the nanocomposites.

The compression–decompression curves for different samples reinforced with the same amount of microfibre, Tch, in combination with different quantities of NKP are shown in Figure 7b. With the increase of the NKP amount, it was observed that the samples become more rigid (the value of Young’s modulus increases) and they support more stress (Table 2). This is due to the increase in density observed when increasing the nanofibres amount (Table 1) and is in agreement with Ref. [38] where the aerogels with lower density offered less resistance to compression stress, while those with higher densities presented stiffer behaviour.

The combination of NKP with microfibres led to an increase of up to three times the mechanical resistance of the nanocomposites when submitted to 25% strain, compared with the systems containing only microfibres. This is because the nanofibres, being smaller, are more dispersed and randomly oriented and, thus, are always compensating for mechanical resistance. Comparing the Young modulus, it is clear that the NKP and Teijinconex^®^ combination (0.817 MPa) is more rigid, compared with NKP and Technora^®^-based system (0.294 MPa).

The recovery after 25% strain varies from 92.7 to 99.8%, similar to that in [26,35]. Generally, the systems with Teijinconex^®^ seem to recover more easily compared to those containing combinations of NKP and Technora^®^. There is a certain influence of the microfibres and nanofibres, being that the recovery of the nanocomposites increases with the increase of the quantity of both nano- and microfibres.

Finally, it is possible to conclude that with the incorporation of the nanofibres, it is possible to improve the mechanical properties of the aerogel nanocomposites reinforced only with aramid fibres, which become more rigid and support more stress.

### 2.3. Assessment of Practical Application of the Nanocomposites

The development of scaled-up samples with redrawn manufacturing conditions is very important for the product’s market approach, as well as the characterization of the nanocomposites under the proper environment of the intended applications. In this sense, we have tested the nanocomposites here prepared under the simulated conditions for a space environment to investigate their suitability for application as thermal insulators under these circumstances. Additionally, we have prepared large-scale samples using the same procedure as for small aerogels with slightly adapted processing steps (see Section 4.4.).

#### 2.3.1. Compliance with Space Conditions

Representative NKP-silica-based aerogels reinforced with Teij and Tch were assessed for compliance with the application conditions using vacuum outgassing and thermal cycling tests (as described in [12]). The outgassing and thermal cycling results are presented in Table 3; the recovered mass loss (RML) was evaluated by the expression:

RML = TML − WVR(1)

where TML is the total mass loss of the material outgassed and WVR is the mass of water vapour regained by the sample after reconditioning (more details in [12]).

RML values less than 1% indicate the capacity of samples to withstand thermal and vacuum conditions and comply with space materials qualification. The nanocomposites reinforced with Technora^®^ fibres met the required standard, while the Teijinconex^®^ is slightly beyond 1% on the third cycle (Table 3). As can be seen in Figure S4, the samples endure the three-cycle analysis maintaining their monolithicity; they lose ~4% of the initial mass and their thermal conductivity increased by 2 mW m^−1^ K^−1^. These results confirm the potential of these samples for future application in space-related devices.

#### 2.3.2. Scale up of the Nanocomposites

**Figure 8:**
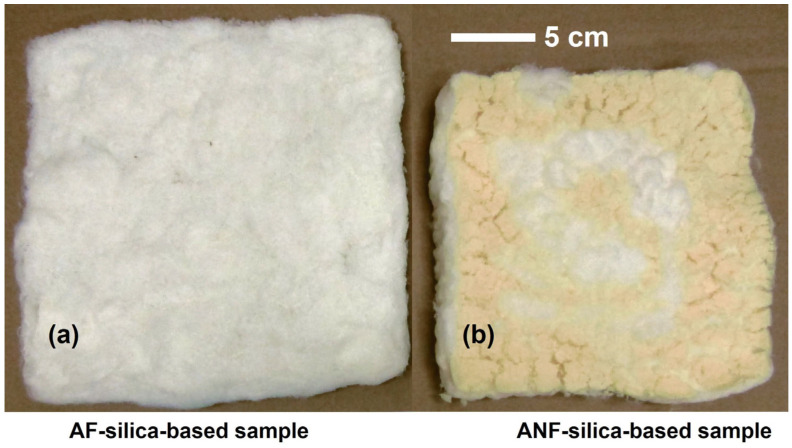
Scaled-up aerogel composites developed with TEOS/VTMS precursors (0.8:0.2) and EtOH/Si molar ratio S = 10 with different reinforcements: (**a**) TV_Teij and (**b**) TV_NKP_Teij. The scale bar is valid for both specimens, manufactured by using the same mould.

There are few works reporting on the preparation of ANF aerogels with large sizes; this is the case of the ANF hydrogel containing reduced graphene oxide and polyaniline [26] in the form of a film with (∼80 × 80 × 0.1 mm^3^) and a polymerization-induced aramid nanofiber (PANF) aerogel with the size of 220 mm × 150 mm × 40 mm [35]. However, none of these mentioned large aerogels were further characterized. Herein, we successfully prepared aerogels with 205 mm × 205 mm × 15 mm (TV_Teij) and 175 mm × 175 mm × 18 mm (TV_NKP_Teij), Figure 8, and their bulk density and thermal conductivity (transient method—Hot Disk and steady-state method—Guarded Hot Plate) were determined.

Both bulk density (171 kg m^−3^—TV_Teij aerogel; 232 kg m^−3^—TV_NKP_Teij aerogel) and thermal conductivity (46.9 ± 2.3 mW m^−1^ K^−1^—TV_Teij aerogel; 55.9 ± 3.6 mW m^−1^ K^−1^—TV_NKP_Teij aerogel) were higher compared to the previous values for smaller samples (153 kg m^−3^ and 32.5 mW m^−1^ K^−1^ for TV_Teij, and 221 kg m^−3^ and 38.3 mW m^−1^ K^−1^ for TV_NKP_Teij). The value of thermal conductivity was lowered by ~8 mW m^−1^ K^−1^ when measured by the guarded hot plate (steady-state) method. An explanation for the less performant features obtained for the larger samples can be the manual deposition of the fibres in the mould that can originate from a lack of homogeneity of the reinforcement matrix, which, in turn, would allow extra paths for heat diffusion through the agglomerated solid matter. In addition, the silylation step could be less efficient, when compared to smaller samples, due to higher diffusion paths.

## 3. Conclusions

Different aerogel nanocomposites reinforced with ANF in combination with AF were synthesized and dried at ambient pressure. The lowest bulk density achieved was 161 kg m^−3^ when combining nanofibres obtained from Kevlar^®^ pulp and Technora^®^ microfibre and the lowest thermal conductivity was 38.3 mW m^−1^ K^−1^ when combining Kevlar^®^ pulp nanofibres and Teijinconex^®^ microfibre. The aerogels with NKP and Teijinconex^®^, as well as with Technora^®^ showed good thermal stability up to 550 °C and exhibited improved mechanical resistance compared with the nanocomposites strengthened with only aramid fibres. The aerogels prepared here present structural, thermal, and mechanical properties similar to or even better than other aerogels, which were prepared by more complex methods. The nanocomposites with nanofibre combinations successfully withstand vacuum and thermal-simulated space conditions and could be easily prepared with larger sizes, therefore representing promising materials for high-temperature thermal-protection systems.

## 4. Materials and Methods

### 4.1. Materials

Tetraethoxysilane (TEOS, 98% purity) and vinyltrimethoxysilane (VTMS, 98% purity) were purchased from Acros Organics; ethanol (purity ≥ 99.8%), n-heptane (purity ≥ 99.5%), and hexamethyldisilazane (HMDZ, 98.5% purity) were obtained from Thermo Scientific; ammonium hydroxide (25% NH_3_ in H_2_O) and oxalic acid anhydrous (purity ≥ 99%) were acquired at Fluka Analytical; dimethylsulfoxide (DMSO, 99.9% purity) was purchased from Fisher Scientific and potassium hydroxide (KOH) was obtained from Laborspirit Lda.

Kevlar^®^ pulp (KP, 0.5–1.0 mm length) was fabricated by *DuPont* (USA) and the aramid fibres with the following trade names: Twaron^®^ (Twa, 100% *para*-aramid fibre, 40–60 mm length); Technora^®^ (Tch, *para-*aramid fibre (co-polymer), 51 mm length) and Teijinconex^®^ (Teij, 100% *meta*-aramid fibre, 51–76 mm length) were kindly offered by Teijin Aramid GmbH (Germany). The main properties of these fibres can be found in Almeida et al. [11].

All reagents were analytical grade and used as received. High-purity water was used to prepare the solutions of oxalic acid (0.01 M) and ammonium hydroxide (1.0 M) catalysts.

### 4.2. Synthesis of Nanofibres

The synthesis of nanofibres was performed through controlled deprotonation in dimethylsulfoxide (DMSO) in the presence of potassium hydroxide (KOH), as reported in [39]. By this procedure, the strength of the hydrogen bonds from amides is reduced, while increasing the repulsive electrostatic forces in the aramid chains, which will finally result in separation (Figure S5a,b); the final solution has different colours, from orange to red, depending on the size of the nanofibres (Figure S5c).

### 4.3. Synthesis of Aerogels

The synthesis of TEOS/VTMS (molar ratio 0.8:0.2) gels was performed using a two-step acid–base catalysed sol-gel process and following the steps illustrated in Figure S6. It was previously observed that the properties of the TEOS-based aerogels were improved when used together with VTMS [32] and VTMS by itself led to aerogels with higher bulk density [40]. The molar ratio of TEOS/VTMS was optimized in [32] using the design of experiments methodology in order to obtain the lowest values for bulk density, thermal conductivity, and Young’s modulus, and the optimum value found was 0.8:0.2. Herein, the precursors were mixed in ethanol (solvent to Si molar ratio: *S* = 10, also optimized in [32]), then oxalic acid (0.01 M) was added to catalyse the hydrolysis and the solution was stirred for 30 min, then placed in an oven at 27 °C. After 16 h, the nanofibers were incorporated in the alcosol, before gelation.

The nanofibers were previously vacuum filtered (from the DMSO solution) using filter paper (Whatman™, 22 µm porosity and 185 µm thickness) after sedimentation in the presence of ethanol. The filtration was only possible for the KP and Tw fibres, for Tch and Teij no filtrate was obtained, probably due to the lower size of these nanofibres (as discussed in Section 2).

The gelation was performed with the addition of ammonium hydroxide (1.0 M) and using an ultrasound bath to ensure a homogeneous mixture, and the gelation time was registered (visual observation of the moment when the viscous sol (fluid) attains certain elasticity able to support stress and stops flowing under the gravitational force [41]). The ageing, solvent exchange, surface modification, and drying steps were performed as reported in [12].

Firstly, aerogels containing only nanofibers were prepared; however, these nanocomposites suffered a high shrinkage during drying and the final nanocomposites were very rigid and cracked easily. In order to reduce the shrinkage and improve the mechanical resistance, a combination with long fibres was investigated. In this sense, aerogel composites were prepared in which the nanofibers and microfibres were introduced alternatively, using a layer-by-layer-like method. The other steps were similarly performed for all nanocomposites.

In addition to aerogels reinforced only with nanofibers, various nanocomposites were prepared (Table S1), including samples containing different amounts of nanofibers and microfibres. Aerogel samples reinforced only with microfibres were also prepared as a reference.

### 4.4. Synthesis of Scaled-Up Samples

Large square samples, 205 mm × 205 mm × 15 mm (TV_Teij) and 175 mm × 175 mm × 18 mm (TV_NKP_Teij) were manufactured with TEOS/VTMS precursors (0.8:0.2), EtOH/Si molar ratio, *S* = 10, by following the two-step procedure previously reported [12]. The total volume of the sol was 1500 mL. These samples were reinforced with either only macrofibres (7.5 g Teijincone*x*^®^ staple fibres), corresponding to the sample TV_Teij_100_, or with a mixture of the same macrofibres with the same amount and nanofibers (~3 g, obtained from Kevlar^®^ pulp using the procedure described in Section 4.2), corresponding to the sample TV_NKP_40__Teij_100_. After the gelation, the procedure was adapted to fit the demand of larger samples. The periods of solvent exchange and silylation (these processes are size deterrent since both diffusion kinetics relates to the gel size [42]) were both extended up to 3 days and the concentration of the silylation solution was increased to 20% (*v*/*v*) HMDZ in heptane. The drying was then performed similarly to that of smaller samples, except for the previous stage of evaporation in the hood which was carried out for ~60 h at ambient temperature, to prevent explosive mixtures of vapour/air during oven drying.

### 4.5. Characterisation of the Nanofibres and Aerogels

The chemical bonding in the nanofibers and aerogels was investigated by ATR-FTIR using an FT/IR4200 (Jasco) in the range 4000 and 550 cm^−1^ with 128 scans and 4 cm^−1^ resolution. The wettability of the aerogel nanocomposites was determined by measuring the contact angle after placing a drop (10 µL) on the surface of the sample, then using ImageJ software to manually measure the angle formed by the tangent to the drop and the surface. Each measurement was performed three times and the value presented is the average of these measurements.

The size of the nanofibers was estimated by dynamic light scattering (DLS) using Zetasizer Nano ZS (Malvern Instruments) with Zetasizer software. For these measurements, the samples were diluted 1000× to avoid interaction between nanofibers and then were placed in a *DTS0012* cuvette. Each experiment consisted of 10 repeated measurements and the value is presented as the average of these measurements. The data processing was performed using the general-purpose distribution, which was indicated by the software as the most appropriate for these dispersions. 

The structure and morphology of the nanofibers and aerogel samples were assessed by scanning electron microscopy (SEM) with a Compact/VP Compact FESEM (ZEISS Merlin) and that of fibres with a TESCAN Vega3 SBH scanning electron microscope. For nanofiber analysis, a certain volume of the dispersion in DMSO was removed and dried under the same conditions as for the aerogels (see [12]). For the analysis of the aerogel samples, thin superficial slices were cut and coated with a thin layer of gold using physical vapour deposition for 30 s. The size of the fibres was determined manually from the SEM images using ImageJ software; the values are given as the average of 10 measurements.

The bulk density (*ρ*_b_) was determined from the ratio of weight to volume, as specified in [32]; the value is given as an average of three sample measurements. The linear shrinkage (Δ*d*/*d*_0_) was calculated using the equation:

Shrinkage (%) = (1 − *d*/*d*_0_) × 100 (2)

where *d*_0_ and *d* is the value of diameter after ageing and after drying.

The porosity of samples can be evaluated from the following equation:

Porosity (%) = (1 − *ρ*_b_/*ρ*_s_) × 100 (3)

where *ρ*_s_ (the skeletal density) was considered to be 1.4 g cm^−3^ based on average values determined in previous work with aerogel composites reinforced with aramid fibres [12].

The thermal stability of aerogels was investigated under an N_2_ atmosphere with a Simultaneous Differential Scanning Calorimeter, DSC/TGA (SDT Q500, TA Instruments) from room temperature (~27 °C) to 800 °C, using a heating rate of 10 °C min^−1^.

The thermal conductivity (*k*) of the aerogels by the transient plane source method was assessed, as in [12], using two sensors: 5501 (diameter = 6.4 mm) and 5465 (diameter = 3.2 mm); the last one for the samples containing only nanofibers, which suffered much shrinkage.

The mechanical properties were investigated with an *Inspekt miniseries* (Hegewald and Peschke) equipment, performing two uniaxial compression–decompression tests at a strain speed of 1 mm min^−1^. One test was carried out with the loading cell of 50 N, up to 25% strain, and then allowed to decompress. This test permitted the evaluation of the Young’s modulus and recovery of the samples. For the second test, a loading cell of 3 kN was used and was performed up to the limit of the cell (destructive test). All tests were performed in duplicate, and the values are given as the average of these measurements.

### 4.6. Compliance with Space Conditions

The compliance of the aerogels with space conditions was performed in the laboratory, reproducing the standard tests, including thermal cycling (ECSS-Q-ST-70-04, ESA, 2008) [43] and outgassing (ECSS-Q-ST-70-02C, ESA, 2008) [44] standard tests. The thermal cycling evaluates the ability of the material to support thermal stress under a space environment, namely its resistance to deleterious effects, such as fracture and cracking, under temperature oscillations within a defined range. The outgassing gives information about the volatile content of materials when exposed to a vacuum environment. The procedures for these tests are as described by Almeida et al. [12].

## Figures and Tables

**Table 1:** Bulk density (***ρ*_b_**), linear shrinkage (Δ*d/d*_0_*)*, porosity (*P*), and thermal conductivity (*k*) for silica aerogel nanocomposites with different reinforcements (nomenclature in Table S1).

System	Δ*d/d*_0_ /%	*ρ*_b_ /kg m^−3^	*P* /%	*k* /mW m^−1^ K^−1^	System	Δ*d/d*_0_ /%	*ρ*_b_ /kg m^−3^	*P* /%	*k* /mW m^−1^ K^−1^
TV_Teij_100_	8.6	153 ± 4	88.8 *	32.5 ± 1.0	TV_Teij_100_	8.6	153 ± 4	88.8 *	32.5 ± 1.0
TV_NKP_25__Teij_50_	35.0	287 ± 7	80.0	47.1 ± 1.3	-	-	-		
TV_NKP_25__Teij_75_	27.8	229 ± 5	83.6	45.9 ± 1.2	-	-	-		
TV_NKP_25__Teij_100_	23.8	212 ±4	84.8	40.2 ± 1.2	TV_NTw_25__Teij_100_	28.7	232 ± 6	83.4	54.6 ± 2.0
TV_NKP_40__Teij_50_	38.9	317 ± 7	77.3	55.2 ± 1.3	-	-	-		
TV_NKP_40__Teij_75_	37.6	298 ± 6	78.7	52.4 ± 1.2	-	-	-		
TV_NKP_40__Teij_100_	26.5	221 ± 4	84.2	38.3 ± 1.1	TV_NTw_40__Teij_100_	29.8	241 ± 5	82.8	48.5 ± 1.5
TV_NKP_50__Teij_100_	27.8	229 ± 5	83.6	44.1 ± 1.3	TV_NTw_50__Teij_100_	28.6	243 ± 5	82.6	52.7 ± 1.8
TV_Tch_100_	6.2	133 ± 3	90.5 *	39.0 ± 1.2	TV_Tch_100_	6.2	133 ± 3	90.5 *	39.0 ± 1.2
TV_NKP_25__Tch_50_	22.4	184 ± 6	86.8	41.9 ± 1.1	-	-	-		
TV_NKP_25__Tch_75_	16.3	170 ± 5	87.8	52.0 ± 1.2	-	-	-		
TV_NKP_25__Tch_100_	16.4	161 ± 4	88.5	51.3 ± 1.2	TV_NTw_25__Tch_100_	19.4	193 ± 3	86.2	60.4 ± 1.5
TV_NKP_40__Tch_50_	32.6	239 ± 2	82.9	53.0 ± 1.4	-	-	-		
TV_NKP_40__Tch_75_	29.5	210 ± 8	85.0	51.2 ± 1.2	-	-	-		
TV_NKP_40__Tch_100_	18.9	175 ± 5	87.5	49.3 ± 1.0	TV_NTw_40__Tch_100_	24.2	198 ± 4	85.8	57.3 ± 1.2
TV_NKP_50__Tch_100_	22.9	182± 5	87.0	52.4 ± 1.2	TV_NTw_50__Tch_100_	27.8	200 ± 5	85.7	58.2 ± 1.0
TV_Tw_100_	7.7	139 ± 3	90.6 *	50.8 ± 1.5	TV_Tw_100_	7.7	139 ± 3	90.6 *	50.8 ± 1.5
TV_NKP_25__Tw_100_	20.6	185 ± 5	86.7	58.7 ± 1.2	TV_NTw_25__Tw_100_	23.2	205 ± 6	85.3	66.2 ± 1.5
TV_NKP_40__Tw_100_	23.6	192 ± 6	86.3	54.5 ± 1.5	TV_NTw_40__Tw_100_	21.6	207 ± 5	85.2	60.8 ± 1.2
TV_NKP_50__Tw_100_	25.8	198 ± 5	85.8	57.0 ± 1.3	TV_NTw_50__Tw_100_	21.9	212 ± 4	84.8	61.1 ± 1.5

* Values from [12].

**Table 2:** Values of Young’s modulus and recovery after 25% strain for silica–aerogel nanocomposites with different reinforcements (nomenclature as in Table S1).

System	*Y*_M_ /kPa	Compressive Stress at 25% Strain /kPa	Recovery /%
TV_Teij_100_	153	53	96.8
TV_NKP_25__Teij_50_	307	100	96.7
TV_NKP_25__Teij_75_	196	52	97.5
TV_NKP_25__Teij_100_	195	46	99.2
TV_NKP_40__Teij_50_	817	461	97.9
TV_NKP_40__Teij_75_	420	122	98.5
TV_NKP_40__Teij_100_	224	63	99.2
TV_NKP_50__Teij_100_	342	175	99.8
TV_Tch_100_	53	21	99.8
TV_NKP_25__Tch_50_	112	47	95.5
TV_NKP_25__Tch_75_	102	44	96.7
TV_NKP_25__Tch_100_	70	30	97.1
TV_NKP_40__Tch_50_	294	163	92.7
TV_NKP_40__Tch_75_	173	54	95.5
TV_NKP_40__Tch_100_	115	35	97.5
TV_NKP_50__Tch_100_	181	62	99.5

**Table 3:** Vacuum outgassing and thermal cycling values for aerogel nanocomposites with different reinforcements (nomenclature as in Table S1).

Test	System	TML %	WVR %	RML %
Vacuum outgassing	TV_NKP_Teij	1.569 ± 0.026	0.752 ± 0.025	0.817 ± 0.051
	TV_NKP_Tch	2.189 ± 0.059	0.497 ± 0.047	1.692 ± 0.012
Thermal cycling	1st cycle			
	TV_NKP_Teij	0.754 ± 0.071	0.009 ± 0.001	0.745 ± 0.070
	TV_NKP_Tch	0.425 ± 0.017	0.004 ± 0.001	0.421 ± 0.016
	2nd cycle			
	TV_NKP_Teij	0.964 ± 0.083	0.008 ± 0.001	0.956 ± 0.082
	TV_NKP_Tch	0.653 ± 0.095	0.005 ± 0.001	0.648 ± 0.094
	3rd cycle			
	TV_NKP_Teij	1.028 ± 0.090	0.009 ± 0.002	1.019 ± 0.088
	TV_NKP_Tch	0.619 ± 0.074	0.489 ± 0.088	0.131 ± 0.014

## Data Availability

The data presented in this study are available on request from the corresponding authors.

## Supplementary Materials

The following supporting information can be downloaded at: https://www.mdpi.com/article/10.3390/gels9070535/s1, Figure S1: Scanning electron microscope images for fibres (left) and nanofibers (right) corresponding to (a) KP, (b) Tw, (c) Teij, and (d) Tch; Figure S2: Influence of the quantity of (a) NKP and (b) NTw on the bulk density of the nanocomposites (nomenclature in Table S1), considering equal quantities of different macrofibres; Figure S3: Stress–strain curves for different systems containing Teij or Tch without or with combination with NKP tested up to a maximum of 3 kN stress; Figure S4: Aerogel nanocomposites based on TEOS/VTMS reinforced with NKP and (A) Teij and (B) Tch tested for thermal cycling, revealing compliance with space applications (subscripts 1 and 2 refer to before and after thermal cycling respectively; Figure S5: Chemical structure of (a) para-aramid and (b) meta-aramid. (c) The visual aspect of the deprotonation solutions of the fibres (from left to right): KP, Tw, Tch, Teij; Figure S6: Schematic representation of the aerogel’s preparation steps; Table S1: Nomenclature and description of the synthesized aerogel samples; Table S2: Gelation time for silica–aerogel nanocomposites based on tetraethoxysilane/vinyltrimethoxysilane (TV) with different reinforcements (nomenclature as in Table S1); Table S3: Comparison with the literature of the bulk density, thermal conductivity and mechanical properties of the silica–aerogel nanocomposites with different reinforcements (nomenclature in Table S1); Table S4: TGA results for fibres and silica–aerogel nanocomposites with different reinforcements (nomenclature as in Table S1).
