# Supplementary material for: Improvement of the Mechanical Properties of Silica Aerogels for Thermal Insulation Applications through a Combination of Aramid Nanofibres and Microfibres
Source: Gels. 2023 Jun 30;9(7):535. doi: 10.3390/gels9070535 (PMC10378766; doi:10.3390/gels9070535)
Supplement: Supplementary file 1 [file gels-09-00535-s001.zip › gels-2414517-supplementary.docx]

**Supporting Information:** **Improvement of the Mechanical Properties of Silica Aerogels for Thermal Insulation Applications through a Combination of Aramid Nanofibres and Microfibres**

Mariana Emilia Ghica *, Jandira G. S. Mandinga, Teresa Linhares, Cláudio M. R. Almeida and Luisa Durães *

University of Coimbra, CIEPQPF, Department of Chemical Engineering, 3030-790 Coimbra, Portugaljandiramandinga2014@outlook.com (J.G.S.M.); tlinhares@eq.uc.pt (T.L.); claudio@eq.uc.pt (C.M.R.A.)

***** Correspondence: meghica@eq.uc.pt (M.E.G.); luisa@eq.uc.pt (L.D.)


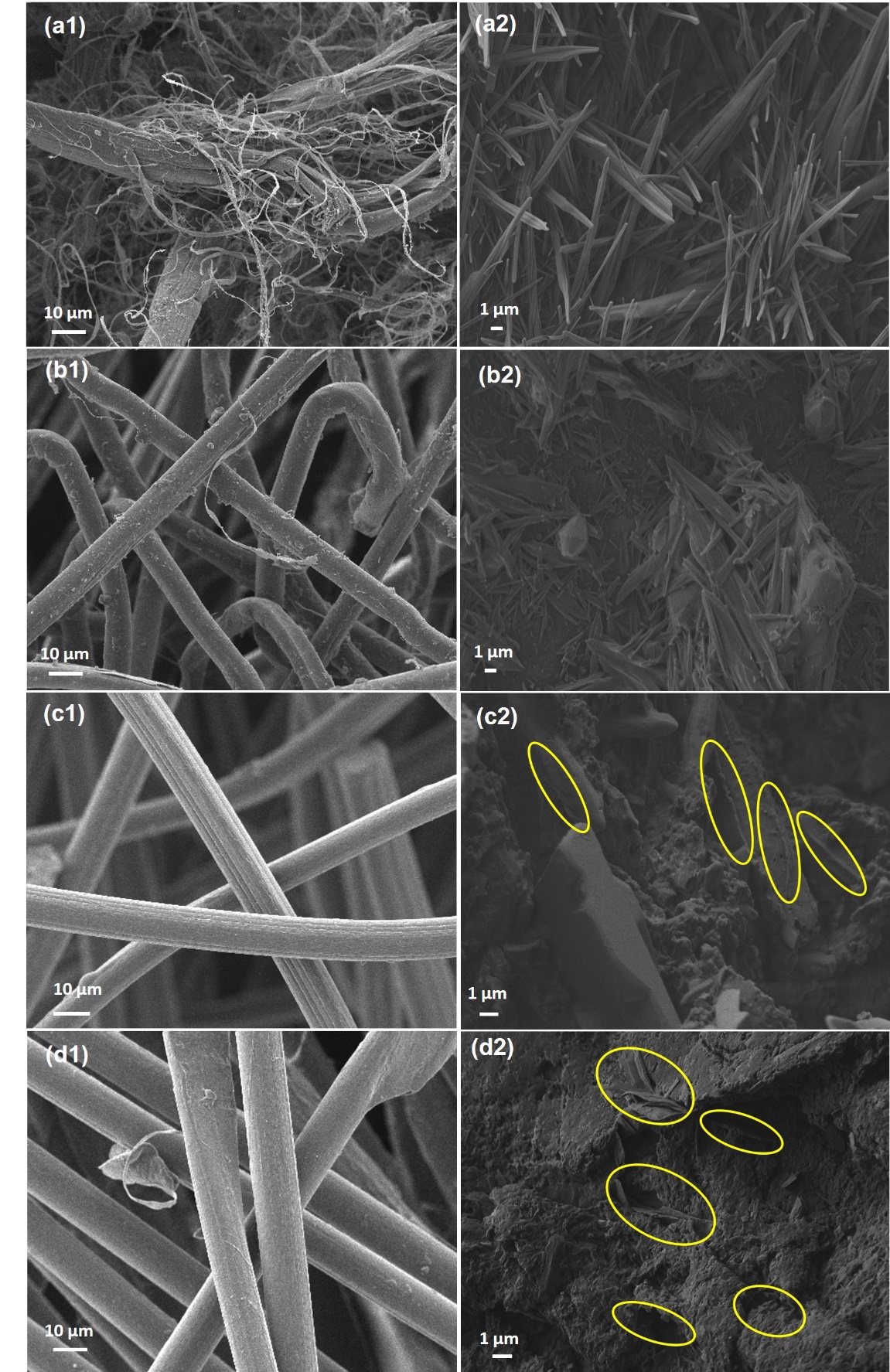


**Figure S1.** Scanning electron microscope images for fibres (left) and nanofibers (right) corresponding to (**a**) KP, (**b**) Tw, (**c**) Teij, (**d**) Tch.

**Table S1.** Nomenclature and description of the synthesized aerogel samples.

| **Nomenclature** | **System Content** |
| --- | --- |
| TV_KP_100_ | (TEOS_0.80_/VTMS_0.20_), KP (100 mg) |
| TV_Tch_100_ | (TEOS_0.80_/VTMS_0.20_), Tch (100 mg) |
| TV_Teij_100_ | (TEOS_0.80_/VTMS_0.20_), Teij (100 mg) |
| TV_Tw_100_ | (TEOS_0.80_/VTMS_0.20_), Tw (100 mg) |
| TV_NKP_50_ | (TEOS_0.80_/VTMS_0.20_), NKP (50 mg) |
| TV_NKP_25__Teij_50_ | (TEOS_0.80_/VTMS_0.20_), NKP (25 mg) + Teij (50 mg) |
| TV_NKP_25__Teij_75_ | (TEOS_0.80_/VTMS_0.20_), NKP (25 mg) + Teij (75 mg) |
| TV_NKP_25__Teij_100_ | (TEOS_0.80_/VTMS_0.20_), NKP (25 mg) + Teij (100 mg) |
| TV_NKP_40__Teij_50_ | (TEOS_0.80_/VTMS_0.20_), NKP (40 mg) + Teij (50 mg) |
| TV_NKP_40__Teij_75_ | (TEOS_0.80_/VTMS_0.20_), NKP (40 mg) + Teij (75 mg) |
| TV_NKP_40__Teij_100_ | (TEOS_0.80_/VTMS_0.20_), NKP (40 mg) + Teij (100 mg) |
| TV_NKP_50__Teij_100_ | (TEOS_0.80_/VTMS_0.20_), NKP (50 mg) + Teij (100 mg) |
| TV_NKP_25__Tch_50_ | (TEOS_0.80_/VTMS_0.20_), NKP (25 mg) + Tch (50 mg) |
| TV_NKP_25__Tch_75_ | (TEOS_0.80_/VTMS_0.20_), NKP (25 mg) + Tch (75 mg) |
| TV_NKP_25__Tch_100_ | (TEOS_0.80_/VTMS_0.20_), NKP (25 mg) + Tch (100 mg) |
| TV_NKP_40__Tch_50_ | (TEOS_0.80_/VTMS_0.20_), NKP (40 mg) + Tch (50 mg) |
| TV_NKP_40__Tch_75_ | (TEOS_0.80_/VTMS_0.20_), NKP (40 mg) + Tch (75 mg) |
| TV_NKP_40__Tch_100_ | (TEOS_0.80_/VTMS_0.20_), NKP (40 mg) + Tch (100 mg) |
| TV_NKP_50__Tch_100_ | (TEOS_0.80_/VTMS_0.20_), NKP (50 mg) + Tch (100 mg) |
| TV_NKP_25__Tw_100_ | (TEOS_0.80_/VTMS_0.20_), NKP (25 mg) + Tw (100 mg) |
| TV_NKP_40__Tw_100_ | (TEOS_0.80_/VTMS_0.20_), NKP (40 mg) + Tw (100 mg) |
| TV_NKP_50__Tw_100_ | (TEOS_0.80_/VTMS_0.20_), KP (50 mg) + Tw (100 mg) |
| TV_NTw_50_ | (TEOS_0.80_/VTMS_0.20_), NTw (50 mg) |
| TV_NTw_25__Teij_100_ | (TEOS_0.80_/VTMS_0.20_), NTw (25 mg) + Teij (100 mg) |
| TV_NTw_40__Teij_100_ | (TEOS_0.80_/VTMS_0.20_), NTw (40 mg) + Teij (100 mg) |
| TV_NTw_50__Teij_100_ | (TEOS_0.80_/VTMS_0.20_), NTw (50 mg) + Teij (100 mg) |
| TV_NTw_25__Tch_100_ | (TEOS_0.80_/VTMS_0.20_), NTw (25 mg) + Tch (100 mg) |
| TV_NTw_40__Tch_100_ | (TEOS_0.80_/VTMS_0.20_), NTw (40 mg) + Tch (100 mg) |
| TV_NTw_50__Tch_100_ | (TEOS_0.80_/VTMS_0.20_), NTw (50 mg) + Tch (100 mg) |
| TV_NTw_25__Tw_100_ | (TEOS_0.80_/VTMS_0.20_), NTw (25 mg) + Tw (100 mg) |
| TV_NTw_40__Tw_100_ | (TEOS_0.80_/VTMS_0.20_), NTw (40 mg) + Tw (100 mg) |
| TV_NTw_50__Tw_100_ | (TEOS_0.80_/VTMS_0.20_), NTw (50 mg) + Tw (100 mg) |

**Table S2.** Gelation time for silica aerogel nanocomposites based on tetraethoxysilane/vinyltrimethoxysilane (TV) with different reinforcements (nomenclature in Table S1).

| **System** | **t_gel_ / min** | **System** | **t_gel_ / min** |
| --- | --- | --- | --- |
| TV_NKP_25__Teij_50_ | 17 | - | - |
| TV_NKP_25__Teij_75_ | 26 | - | - |
| TV_NKP_25__Teij_100_ | 28 | TV_NTw_25__Teij_100_ | 16 |
| TV_NKP_40__Teij_50_ | 17 | - | - |
| TV_NKP_40__Teij_75_ | 26 |  | - |
| TV_NKP_40__Teij_100_ | 21 | TV_NTw_40__Teij_100_ | 14 |
| TV_NKP_50__Teij_100_ | 11 | TV_NTw_50__Teij_100_ | 9 |
| TV_NKP_25__Tch_50_ | 12 | - | - |
| TV_NKP_25__Tch_75_ | 15 | - | - |
| TV_NKP_25__Tch_100_ | 22 | TV_NTw_25__Tch_100_ | 14 |
| TV_NKP_40__Tch_50_ | 16 | - | - |
| TV_NKP_40__Tch_75_ | 20 | - | - |
| TV_NKP_40__Tch_100_ | 15 | TV_NTw_40__Tch_100_ | 12 |
| TV_NKP_50__Tch_100_ | 12 | TV_NTw_50__Tch_100_ | 10 |
| TV_NKP_25__Tw_100_ | 16 | TV_NTw_25__Tw_100_ | 15 |
| TV_NKP_40__Tw_100_ | 10 | TV_NTw_40__Tw_100_ | 12 |
| TV_NKP_50__Tw_100_ | 9 | TV_NTw_50__Tw_100_ | 12 |


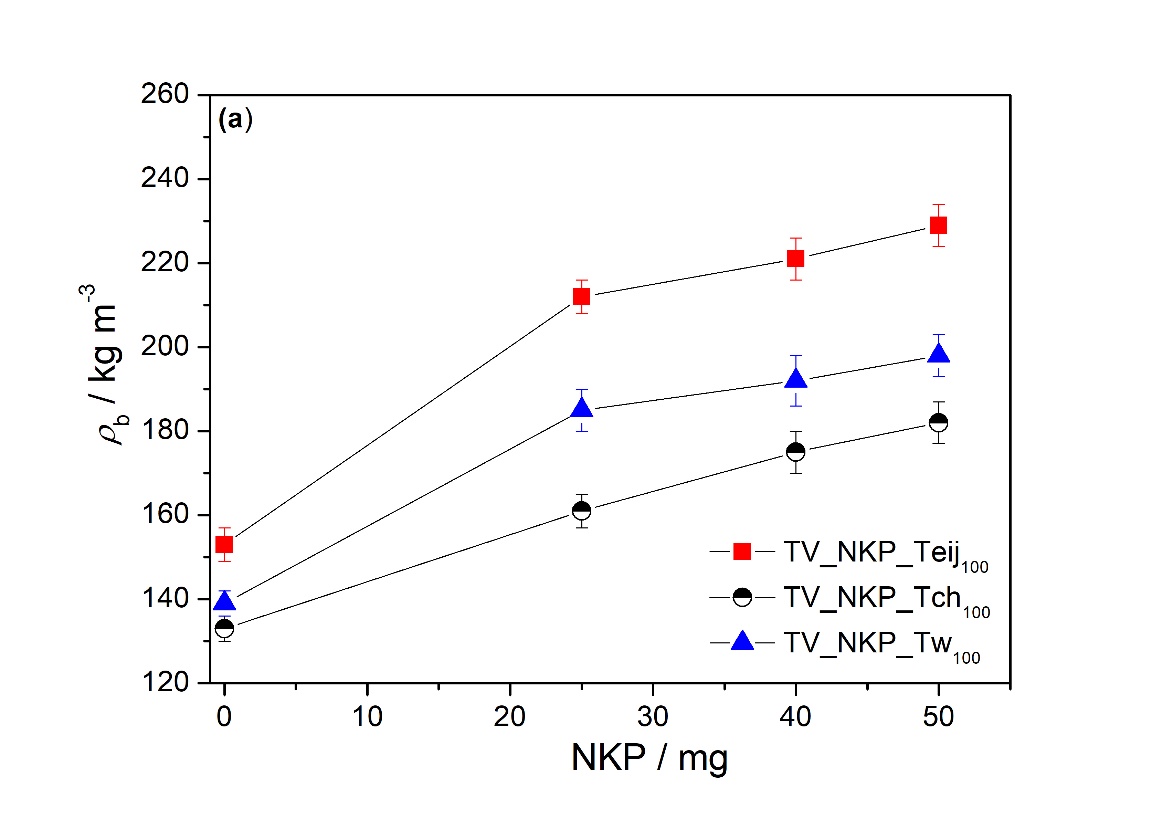


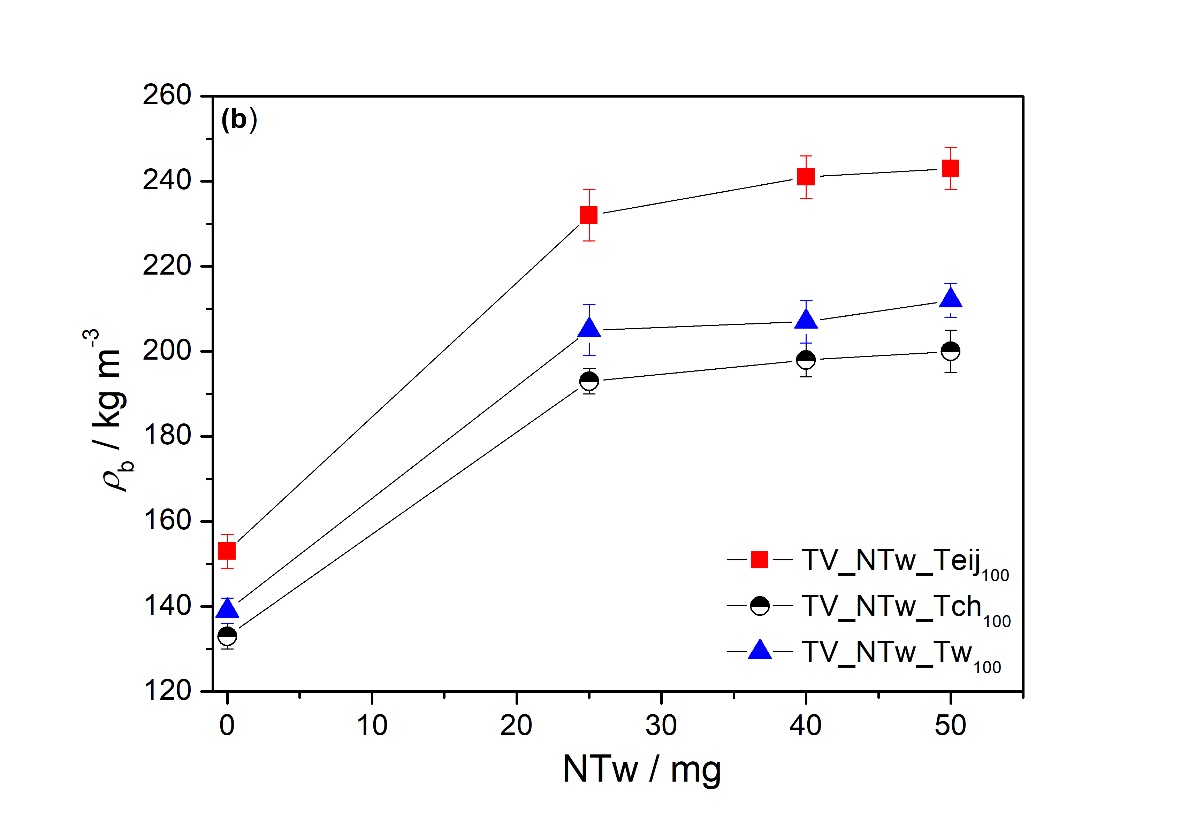


**Figure S2.** Influence of the quantity of (**a**) NKP and (**b**) NTw on the bulk density of the nanocomposites (nomenclature in Table S1), considering equal quantities of different microfibres.

**Table S3**. Comparison with literature of the bulk density, thermal conductivity and mechanical properties of the silica aerogel nanocomposites with different reinforcements (nomenclature in Table S1).

| **System** | **Drying Method** | ***ρ*_b_ / kg m^-3^** | ***k*  / mW m^-1^ K^-1^** | **Compressive Strength at 50% stress / kPa** | **Reference** |
| --- | --- | --- | --- | --- | --- |
| TEOS_ATPNF | APD | 163-192 | 19.8-22.8 | 800-2500 | [5] |
| TEOS_AP | APD | 150.8-161.5 | 23.2-27.8 | 830-1220 | [7] |
| TEOS_NCF | CO_2_-SCD | 70-83 | 11.3-12.1 | 140 | [11] |
| ANAFs | WS/FD | 24-114 | 31-34.0 | ND | [23] |
| ANFAs | VFD/VH | 1.42-5.20 | 23.7-26.3 | 2.5-2.9 | [24] |
| ANFs/TEOS | CO_2_-SCD/CVD | 96.9-298.8 | 30.2-54.2 | ND | [28] |
| PANFA | FD | 20-185 | 33.9-71.8 | 60-1800 | [35] |
| HDPISG | F/VD/VH | 20-280 | 41.1-89.3 | 6-5190 | [36] |
| PIFA | FE/VD | 39.1-52.8 | 40.4-44.0 | 49-189 | [37] |
| TV_Teij | APD | 153 | 32.5 | 441 | [12] |
| TV_Tch | APD | 133 | 39.0 | 257 | [12] |
| TV_NKP_Tch | APD | 161-239 | 41.9-53.0 | 1080 | This work |
| TV_NKP_Teij | APD | 212-317 | 38.3-55.2 | 1960 | This work |

TEOS – tetraethoxysilane; ATPNF – attapulgite nanofibre; AP – aramid pulp; NCF –cellulose nanofibres; ANAFs – aramid nanofibre aerogel fibres; ANFAs – aramid nanofibre aerogels; PANFA – polymerisation-induced aramid nanofibre aerogel; HDPISG – high density polyimide sponges; PIFA – polyimide fibrous aerogels; APD – ambient pressure drying; CO_2_-SCD – CO_2_ supercritical drying; WS/FD – wet spinning/freeze drying; VFD/VH – vacuum freezing/vacuum heating; CVD – chemical vapour deposition; F/VD/VH – freezing/vacuum drying/vacuum heating; FE/VD – freeze-extraction/vacuum-drying; ND – not determined.

**Table S4.** TGA results for fibres and silica aerogel nanocomposites with different reinforcements (nomenclature as in Table S1).

| **Sample** | ***T*_onset_**  **^ᴏ^C** | ***T*_end_**  **^ᴏ^C** | **Weight loss**  **%** | **Degradation /Evaporation** | **Weight loss % at 550 °C** | **Residue**  **%** |
| --- | --- | --- | --- | --- | --- | --- |
| **KP** | 23.1 | 57.1 | 4.7 | H_2_O | 11.9 | 34.9 |
|  | 559.6 | 588.7 | 54.8 | KP |  |  |
| **NKP** | 42.9 | 72.1 | 24.4 | H_2_O | 51.5 | 30.4 |
|  | 89.4 | 98.6 | 4.6 | H_2_O |  |  |
|  | 277.1 | 307.8 | 3.8 | DMSO |  |  |
|  | 398.7 | 414.0 | 6.6 | 1^st^ stage NKP |  |  |
|  | 496.6 | 533.8 | 4.3 | 2^nd^ stage NKP |  |  |
| **Teij** | 23.7 | 79.4 | 3.8 | H_2_O | 33.9 | 52.4 |
|  | 293.9 | 332.2 | 1.4 | by-products |  |  |
|  | 421.7 | 464.9 | 15.8 | 1^st^ stage Teij |  |  |
|  | 505.6 | 572.6 | 20.2 | 2^nd^ stage Teij |  |  |
| **Tch** | 51.1 | 96.5 | 1.1 | H_2_O | 34.8 | 47.3 |
|  | 486.3 | 509.0 | 18.7 | 1^st^ stage Tch |  |  |
|  | 533.4 | 573.1 | 27.6 | 2^nd^ stage Tch |  |  |
| **TV_KP** | 56.3 | 82.3 | 0.1 | EtOH/Heptane/H_2_O | 10.2 | 90.0 |
|  | 495.3 | 598.5 | 9.6 | -CH_3_, CH=CH_2,_ KP |  |  |
| **TV_NKP** | 46.0 | 64.8 | 0.2 | H_2_O/EtOH | 14.2 | 80.7 |
|  | 127.3 | 170.2 | 1.2 | Heptane |  |  |
|  | 240.7 | 327.7 | 5.8 | -OH, DMSO |  |  |
|  | 470.5 | 577.8 | 10.1 | -CH_3_, CH=CH_2_, NKP |  |  |
| **TV_Teij** | 27.6 | 71.6 | 1.1 | EtOH/Heptane/H_2_O | 8.5 | 81.3 |
|  | 290.2 | 354.6 | 2.8 | -OH |  |  |
|  | 425.1 | 467.5 | 3.9 | -CH_3_/1^st^ stage Teij |  |  |
|  | 515.1 | 592.5 | 8.5 | CH=CH_2_/2^nd^ stage Teij |  |  |
| **TV_Tch** | 52.7 | 86.9 | 0.4 | EtOH/Heptane/H_2_O | 6.6 | 77.2 |
|  | 485.7 | 563.9 | 19.0 | -CH_3_/CH=CH_2_/Tch |  |  |
| **TV_NKP___Teij** | 103.5 | 154.8 | 2.9 | EtOH/Heptane/H_2_O | 17.9 | 76.4 |
|  | 237.5 | 297.1 | 2.6 | -OH, DMSO |  |  |
|  | 430.3 | 475.2 | 6.0 | -CH_3_, NKP, Teij |  |  |
|  | 509.9 | 581.2 | 7.8 | CH=CH_2_, NKP, Teij |  |  |
| **TV_NKP___Tch** | 106.2 | 153.2 | 1.6 | EtOH/Heptane/H_2_O | 13.7 | 81.6 |
|  | 213.7 | 345.8 | 6.2 | -OH, DMSO |  |  |
|  | 467.9 | 503.3 | 3.8 | -CH_3_, NKP, Tch |  |  |
|  | 521.3 | 588.2 | 4.7 | CH=CH_2_, NKP, Tch |  |  |


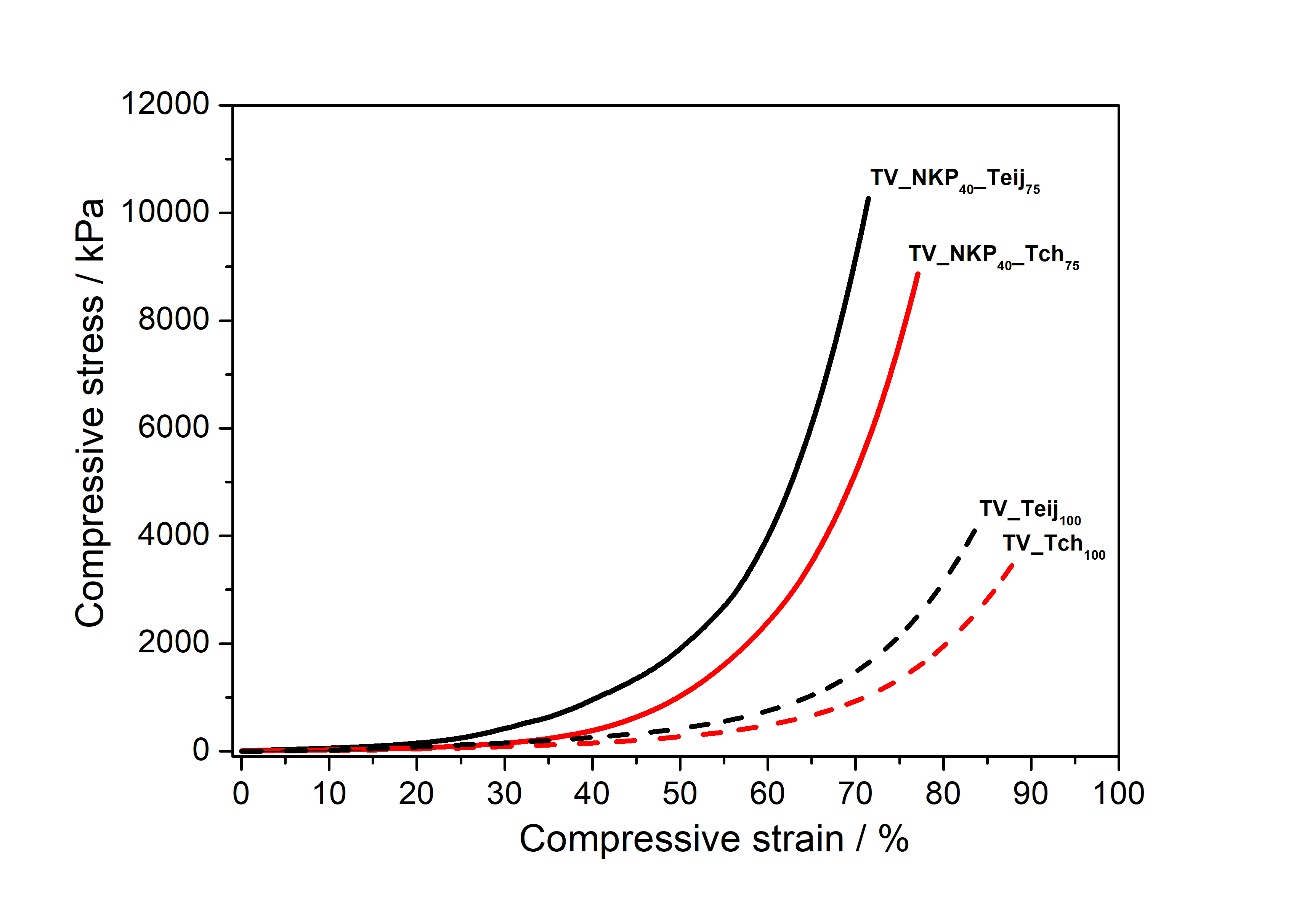


**Figure S3**. Stress-strain curves for different systems containing Teij or Tch without or with combination with NKP and tested up to maximum of 3 kN stress.


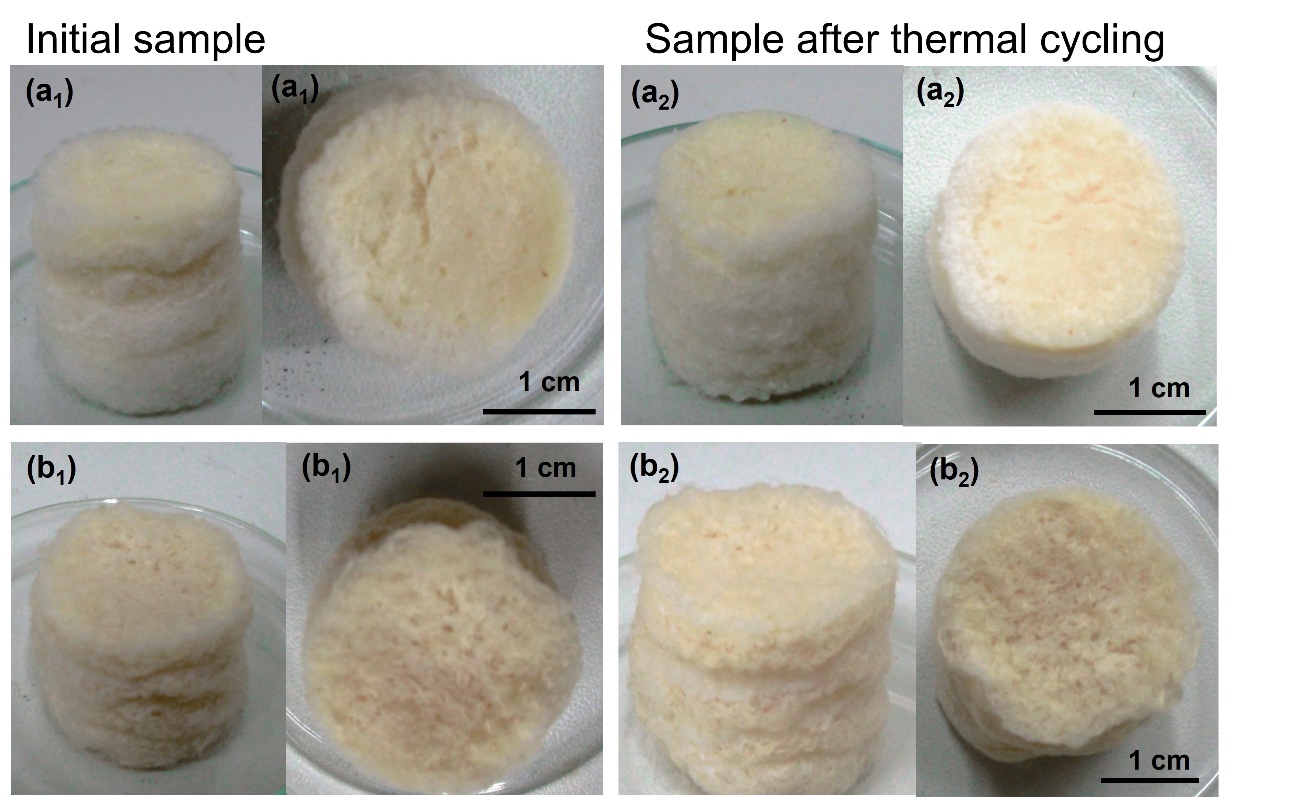


**Figure S4.** Aerogel nanocomposites based on TEOS/VTMS reinforced with NKP and (**a**) Teij and (**b**) Tch tested for thermal cycling, revealing compliance with Space applications (subscripts 1 and 2 refer to before and after thermal cycling respectively).

*Synthesis of Nanofibres*

Briefly, the preparation consisted in weighting 1 g of fibre and 1.5 g of KOH finely grounded and adding into 500 mL of DMSO. The mixture was stirred at room temperature for one week until an orange/red colour solution was formed (depending on fibres´ size) (Figure S5c).


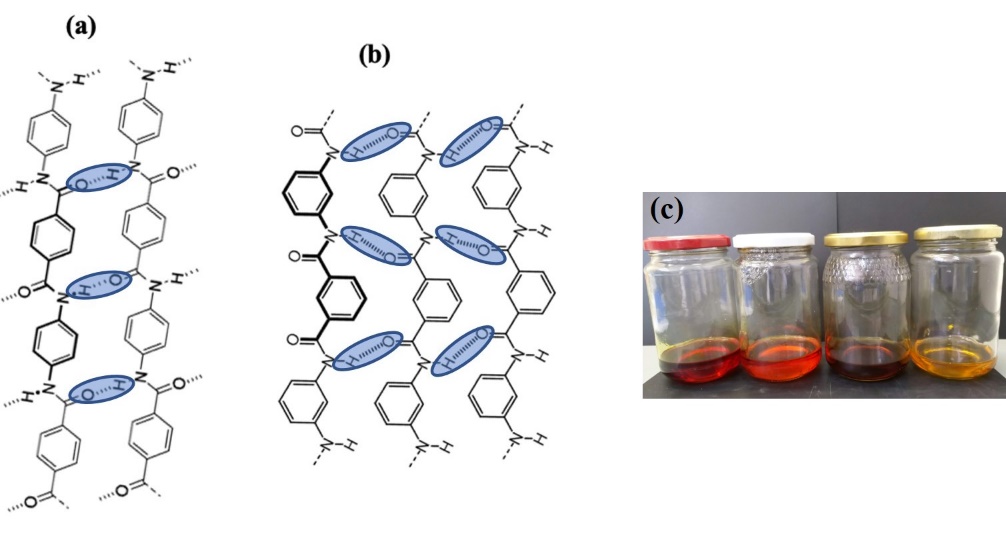


**Figure S5.** Chemical structure of (**a**) *para*-aramid and (**b**) *meta*-aramid. (**c**) Visual aspect of the deprotonation solutions of the fibres (from left to right): KP, Tw, Tch, Teij.


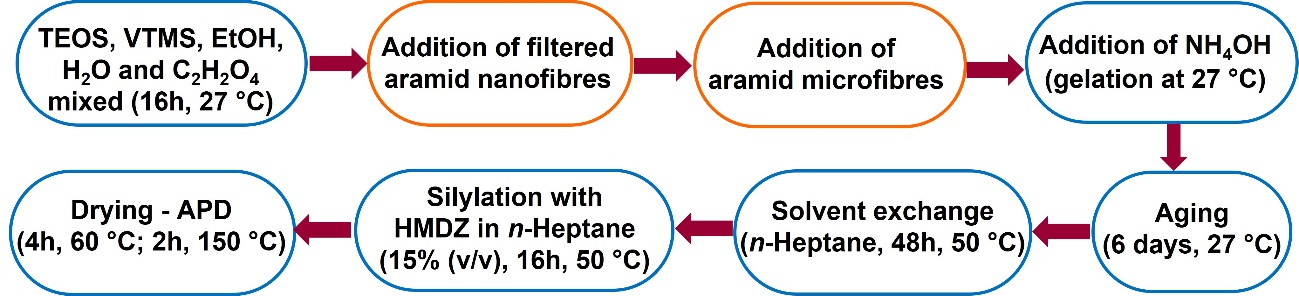


**Figure S6.** Schematic representation of the aerogel’s preparation steps.
